# Association between hypotension and myocardial injury in patients with severe trauma

**DOI:** 10.1007/s00068-022-02051-5

**Published:** 2022-08-03

**Authors:** Alexandra Stroda, Simon Thelen, René M’Pembele, Nick Khademlou, Carina Jaekel, Erik Schiffner, Dan Bieler, Michael Bernhard, Ragnar Huhn, Giovanna Lurati Buse, Sebastian Roth

**Affiliations:** 1 Department of Anesthesiology, Medical Faculty and University Hospital Duesseldorf, Heinrich-Heine-University Duesseldorf, Moorenstr. 5, 40225 Duesseldorf, Germany; 2 Department of Orthopedics and Trauma Surgery, Medical Faculty and University Hospital Duesseldorf, Heinrich-Heine-University Duesseldorf, Moorenstr. 5, 40225 Duesseldorf, Germany; 3 Emergency Department, Medical Faculty and University Hospital Duesseldorf, Heinrich-Heine-University Duesseldorf, Moorenstr. 5, 40225 Duesseldorf, Germany; 4 Department of Anesthesiology, Kerckhoff Heart and Lung Center, Bad Nauheim, Germany

**Keywords:** Troponin, Blood pressure, Multiple trauma, Hemodynamics, Permissive hypotension, Mean arterial pressure

## Abstract

**Purpose:**

During resuscitation of patients with severe trauma, guidelines recommend permissive hypotension prior to surgical bleeding control. However, hypotension may be associated with reduced organ perfusion and multiple organ dysfunction, e.g. myocardial injury. The association between hypotension and myocardial injury in trauma patients is underexplored. We hypothesized that hypotension is associated with myocardial injury in this population.

**Materials and methods:**

This retrospective study included patients ≥ 18 years suffering from severe trauma [defined as Injury Severity Score (ISS) ≥ 16] that were treated in the emergency department resuscitation room between 2016 and 2019. Primary endpoint was the incidence of myocardial injury defined as high-sensitive troponin T > 14 ng/l. Main exposure was the duration of arterial hypotension during resuscitation period defined as mean arterial pressure < 65 mmHg.

**Results:**

Out of 368 patients screened, 343 were analyzed (73% male, age: 55 ± 21, ISS: 28 ± 12). Myocardial injury was detected in 143 (42%) patients. Overall in-hospital mortality was 26%. Multivariate binary logistic regression with forced entry of nine predefined covariables revealed an odds ratio of 1.29 [95% confidence interval 1.16–1.44]; *p* = 0.012) for the association between the duration of hypotension and myocardial injury.

**Conclusion:**

The duration of hypotension during resuscitation period is independently associated with the incidence of myocardial injury in patients with severe trauma.

**Supplementary Information:**

The online version contains supplementary material available at 10.1007/s00068-022-02051-5.

## Introduction

During resuscitation of patients suffering from multiple injuries and consecutive hemorrhagic shock, current guidelines recommend—after the exclusion of neurotrauma—fluid volume restriction and permissive hypotension prior to surgical bleeding control [1]. The aim is to prevent coagulopathy and hydrostatic pressure on the wound [2]. However, permissive hypotension may be associated with reduced organ perfusion and may lead to multiple organ dysfunction [3]. In the non-cardiac surgery setting, there is accumulating evidence that perioperative hypotension is associated with myocardial injury [4, 5]. This entity is defined by any detection of troponin exceeding the 99^th^ percentile, independent of its pathophysiological mechanism [6]. Myocardial injury is associated with higher mortality and an increased rate of major adverse cardiovascular events (MACE) after non-cardiac surgery [7, 8]. Also in the trauma setting, myocardial injury was common and independently associated with mortality [9]. In the trauma population, the association between hypotension and myocardial injury is still underexplored. We hypothesized that hypotension, defined as a mean arterial blood pressure < 65 mmHg, is associated with myocardial injury in patients suffering from severe trauma.

## Methods

We conducted a retrospective, single-center cohort study that follows the guidelines for good clinical practice (GCP) and was conducted in accordance with the declaration of Helsinki. The study was approved by the ethical committee of Heinrich-Heine-University Dusseldorf, Germany (Reference Number 2020–1122). All handling of personal data followed the General Data Protection Regulation (EU) 2016/679 and complied with the GCP Guidelines. The structure of this manuscript complies with the STROBE reporting guidelines for retrospective cohort studies.

### Participants

Inclusion criteria were as follows: severely injured (Injury Severity Score (ISS) ≥ 16), adult (≥ 18 years) patients that were admitted to the emergency department (ED) resuscitation room of the University Hospital Dusseldorf, Germany, between 2016 and 2019. Patients were excluded if (1) they were declared dead immediately after arrival, (2) no measurement of troponin T at arrival at the resuscitation room was available or (3) if blood pressure values were missing (defined as no documented blood pressure measurement for more than 5 min).

### Exposures and primary endpoint

The main exposure was the relative duration of arterial hypotension, defined as the time of mean arterial pressure < 65 mmHg during resuscitation period divided by the total time spent in the resuscitation room. Blood pressure was measured according to standard care, i.e. non-invasive measurement was implemented immediately after ED arrival, with blood pressure being recorded at least every minute. As soon as possible, invasive and continuous arterial blood pressure measurement was initiated. Blood pressure values were automatically transmitted from the monitor to a digital documentation program (COPRA^®^, COPRA System GmbH, Berlin, Germany) and later extracted to the study database.

The primary endpoint was myocardial injury as defined by the fourth universal definition of myocardial infarction, detected using high-sensitivity troponin T (hsTnT, Roche Diagnostics, Elecsys^®^, Rotkreuz, Switzerland) [6]. HsTnT was collected shortly after arrival to the resuscitation room as part of the standard protocol for severe trauma and immediately analyzed at the hospital central laboratory. Troponin concentrations were later extracted from clinical documentation for this study.

Importantly, measurements of blood pressure and hsTnT were performed simultaneously which carries the risk that there were events or interventions in the course of resuscitation period that could not be captured by initial hsTnT measurements. For this reason, an additional analysis using myocardial injury based on peak hsTnT measurement within 72 h after trauma was performed. Due to a higher risk for selection bias, peak hsTnT was determined to be a secondary *endpoint*.

### Further secondary endpoints

Further secondary endpoints were in-hospital mortality, in-hospital major adverse cardiovascular events (MACE, defined as non-fatal cardiac arrest, acute myocardial infarction, new cardiac arrhythmias including atrial fibrillation or stroke) and acute kidney injury (AKI) within 72 h after ED arrival defined by the Kidney Disease Improving Global Outcomes (KDIGO) criteria. This information was extracted from clinical documentation by trained personnel.

### Statistical analysis

Complete case analysis was conducted. Categorical data are presented as absolute numbers (percent). Continuous data are presented as mean (standard deviation) or median (interquartile range) as applicable. The level of significance was at two-tailed *p* ≤ 0.05. SPSS^®^ 25 was used as statistical software. We quantified the association between hypotension and the primary endpoint by multivariate binary logistic regression with forced entry of predefined covariables (see below). Further we calculated the area under the curve (AUCs) for the logistic regression models with and without duration of hypotension to determine potential improvement and performed De Long-Test for comparison of AUCs. Hosmer–Lemeshow test was performed to evaluate goodness of fit.

We predefined covariables for multivariate regression analysis. The following covariables were chosen for multivariable adjustment as they showed potential impact on myocardial injury and/or hypotension in the literature [10–12]: age (continuous), sex, ISS (continuous), pre-existing chronic kidney injury (as defined by KDIGO Criteria), pre-existing coronary artery disease, thorax trauma, out-of-hospital resuscitation and hemoglobin (continuous). Due to the retrospective nature of the study, we did not conduct a formal sample size calculation. In presence of 143 events, a maximum of 14 covariables could be included into multivariate analysis [13]. For the other logistic regression models (MACE, AKI and mortality), the same covariables were chosen.

As already mentioned above, we performed additional analysis, taking into account, that there were events or interventions in the course of resuscitation period that could not be captured by initial hsTnT measurements: (1) We performed a sensitivity analysis for systolic blood pressure (Sys) < 90 as alternative exposure. (2) We performed multivariate logistic regression for the absolute duration of hypotension instead of the relative duration of hypotension during resuscitation period for MAP < 65 mmHg and Sys < 90 mmHg. (3) We performed multivariate logistic regression analysis for the association between hypotension and myocardial injury using preclinical covariables. 4) We used myocardial injury based on peak hsTnT measurement within the first 72 h after trauma as an alternative endpoint for all performed multivariate logistic regression models.

## Results

**Fig. 1:**
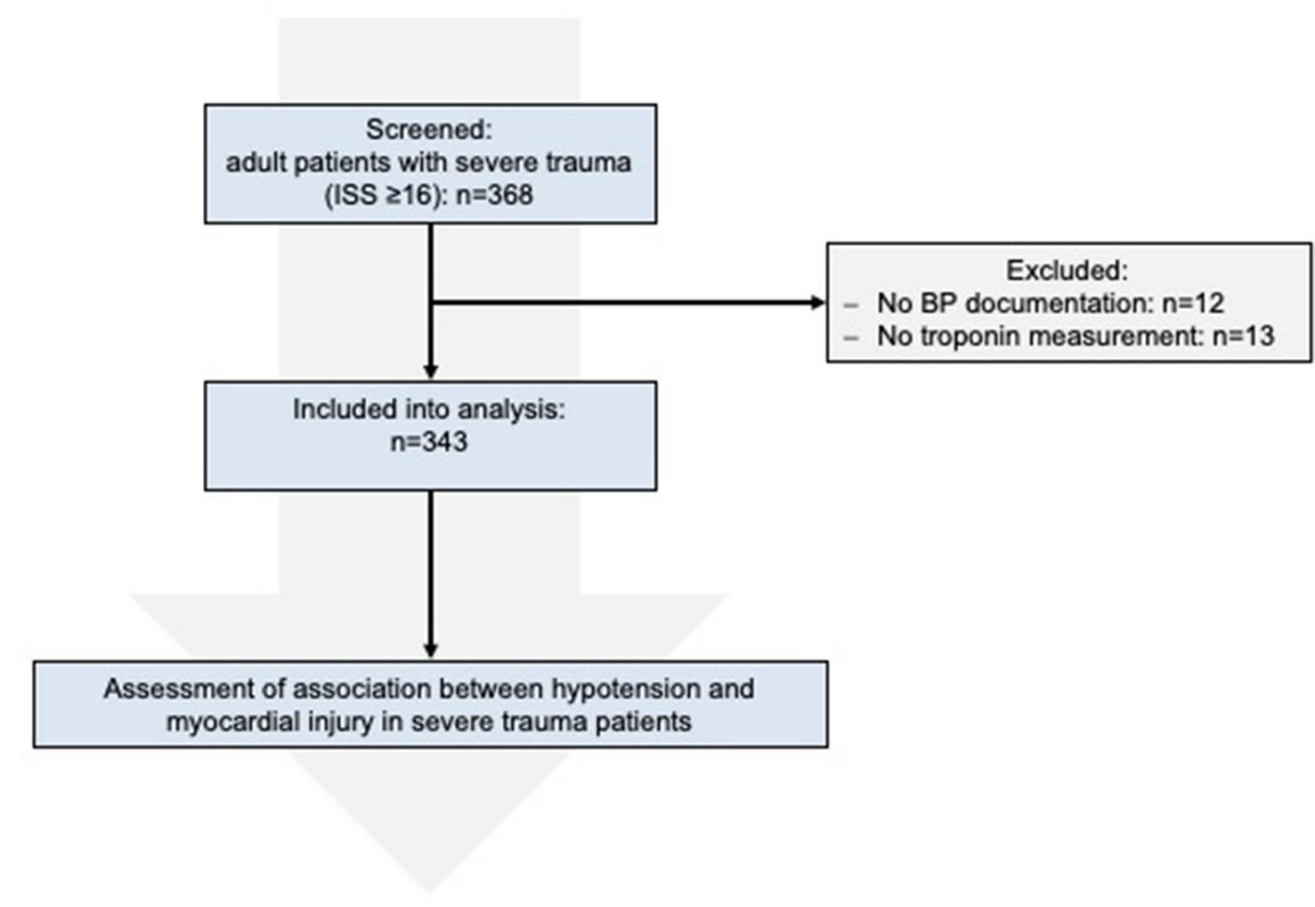
Study flowchart

Of 368 screened patients, 343 (93%) were included into the analysis (Fig. 1). Patient characteristics are presented in Table 1. Mean proportion of duration of arterial hypotension was 15% ± 23%. Median proportion of duration of arterial hypotension during resuscitation period was 0% [0–22%] which means that 25% of patients had hypotension for more than 20% of the total time spent in the resuscitation room. Mean hsTnT was 59 ± 418 ng/ml and 143 (41.7%) patients presented myocardial injury (hsTnT > 14 ng/l). In-hospital mortality was 25.7% (88/343). In-hospital MACE was detected in 9.0% (31/343) and the incidence of AKI was 11.7% (40/343). At least one follow-up hsTnT measurement was available in 190 (55.4%) patients and 131 (68.9%) out of these patients had myocardial injury based on peak hsTnT within this time frame.

**Table 1:** Patient characteristics

	Patients with severe trauma (*n* = 343)	Patients with myocardial injury (*n* = 143)	Patients without myocardial injury (*n* = 200)
Baseline characteristics			
Male sex no. (%)	250 (71.0%)	102 (71.3%)	148 (74.0%)
Age (years)	55 ± 21	62 ± 23	50 ± 18
Adipositas (body mass index ≥ 30 kg/m^2^)	3 (0.8%)	2 (1.4%)	1 (0.5%)
Comorbidities			
Coronary artery disease	26 (7.6%)	15 (10.5%)	11 (5.5%)
Chronic kidney disease (≥ CKD III)	7 (2.0%)	6 (4.2%)	1 (0.5%)
Diabetes mellitus	21 (6.1%)	8 (5.6%)	13 (6.5%)
History of arterial hypertension	82 (23.9%)	39 (27.3%)	43 (21.5%)
ASA physical status			
ASA I	156 (48.6%)	47 (36.4%)	109 (56.8%)
ASA II	108 (33.6%)	54 (41.9%)	54 (28.1%)
ASA III	50 (15.6%)	23 (17.8%)	27 (14.1%)
ASA IV	7 (2.2%)	5 (3.9%)	2 (1.0%)
Trauma related data			
ISS	28 ± 12	31 ± 1	26 ± 10
Penetrating trauma	15 (4.4%)	5 (3.5%)	10 (5.0%)
Fall < 3 m	82 (23.9%)	47 (33.1%)	35 (17.9%)
Fall > 3 m	85 (24.8%)	36 (25.4%)	49 (25%)
Car-/truck driver	28 (8.1%)	8 (5.6%)	20 (10.2%)
Motorcyclist	29 (8.4%)	9 (6.3%)	20 (10.2%)
Bicyclist	30 (8.7%)	8 (5.6%)	22 (11.2%)
Pedestrian	38 (11.1%)	12 (8.5%)	26 (13.3%)
Traffic other	6 (1.7%)	3 (2.1%)	3 (1.5%)
Stitch damage	10 (2.9%)	4 (2.8%)	6 (3.1%)
Hit	8 (2.3%)	4 (2.8%)	4 (2.0%)
Other	22 (6.4%)	11 (7.7%)	11 (5.6%)
GCS at ED arrival	3 [3–14]	3 [3–9]	10 [3–15]
Hypotension MAP (ratio)	0.0 [0.0–0.22]	0.12 [0.0–0.41]	0.0 [0.0–0.14]
Hypotension Sys (ratio)	0.0 [0.0–0.19]	0.11 [0.0–0.24]	0.0 [0.0–0.12]
Thorax trauma	176 (51.3%)	78 (54.4%)	98 (49.0%)
Resuscitation	34 (10.8%)	26 (20.8%)	8 (4.2%)
Laboratory values			
Hb (g/dl)	12.2 ± 2.4	11.5 ± 2.5	12.7 ± 2.2
INR	1.4 ± 0.8	1.5 ± 1.0	1.2 ± 0.5
PTT (sec)	31.5 ± 23.8	37.6 ± 31.6	27.3 ± 15.1
Base excess	− 3.7 ± 5.6	− 5.5 ± 6.4	− 2.5 ± 4.5
HsTnT initial (ng/ml)	59.4 ± 418.4	132.0 ± 642.3	7.44 ± 3.0
Pharmacological therapy			
Tranexamic acid	21 (6.7%)	6 (4.9%)	15 (7.9%)
Vasopressors	72 (21.8%)	47 (37%)	25 (13.3%)
Sedatives	241 (75.8%)	98 (76%)	143 (75.7%)
Blood products			
Erythrocytes	89 (30.2%)	51 (39.2%)	38 (23%)
Fresh frozen plasma	84 (27.3%)	42 (32.3%)	42 (23.6%)
Platelets	38 (13.1%)	24 (19%)	14 (8.5%)
Outcome			
Death in hospital	88 (25.7%)	63 (44.1%)	25 (12.5%)
In-hospital MACE	31 (9.0%)	21 (14.7%)	10 (5.0%)
AKI	40 (11.7%)	26 (18.2%)	14 (7.0%)
Length of hospital stay	13 [5–21]	9 [2–18]	15 [8–23]
Length of ICU stay	3 [1–21]	4 [1–11]	3 [1–31]

*ASA* American society of anesthesiologists, *ISS* injury severity score, *ED* emergency department, *GCS* glassgow coma scale, *Hb* haemoglobin, *INR* International normalized ratio, *PTT* partial thromboplastin time, *HsTnT* high-sensitive troponin, *MACE* major adverse cardiovascular event, *AKI* acute kidney injury, *ICU* intensive care unit

^*^Values are presented as N (%) or Mean (± SD)/Median [IQL], where appropriate

### Association between hypotension and myocardial injury

**Fig. 2:**
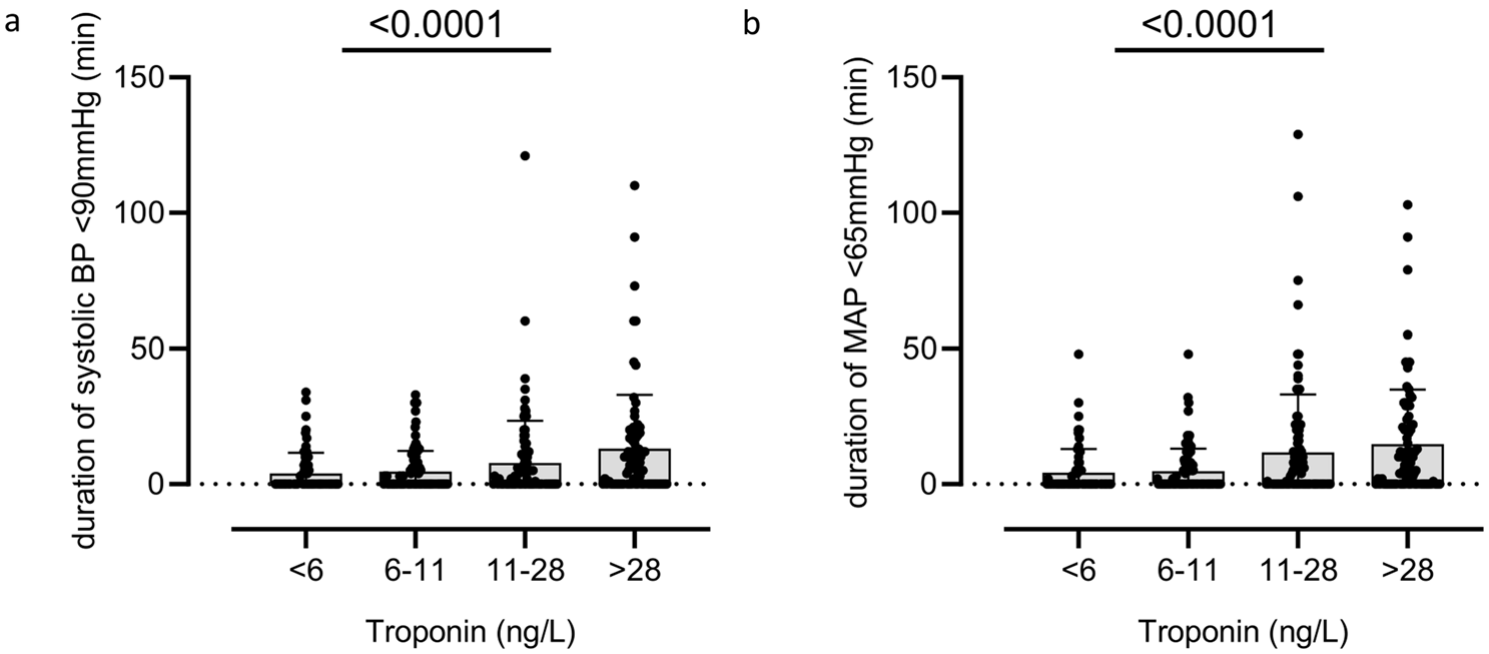
Association between troponin values (presented by quartiles) and the absolute duration of hypotension during resuscitation period in minutes (min). *BP* blood pressure, *MAP *mean arterial pressure

Univariate logistic regression analysis of hypotension duration for myocardial injury showed an odds ratio (OR) of 1.29 [95% confidence interval (CI) 1.16–1.44]. Figure 2 shows an additional univariate analysis of the association between absolute troponin values by quartiles and the absolute duration of hypotension (see Fig. 2). After multivariable adjustment, the association persisted (OR 1.22 [95% CI 1.04–1.42]) (see Table 2). Hosmer–Lemeshow test did not indicate relevant miscalibration (*p* = 0.41). The AUC for the logistic regression model was 0.78 [95% CI 0.72–0.83] including hypotension and 0.75 [95% CI 0.69–0.81] without hypotension. De Long test showed no significant difference between the models. Full results of multivariate analysis are reported in Table 2.

**Table 2:** Multivariate binary logistic regression model for hypotension and myocardial injury

Variable	Regression coefficient	Odds ratio	95% confidence interval		*p*-value
			Lower	Upper	
Hypotension*	0.197	1.218	1.044	1.419	0.012
Age per year	0.035	1.035	1.020	1.051	< 0.001
Sex	0.195	1.215	0.644	2.293	0.547
ISS-score	0.006	1.006	0.980	1.033	0.661
Coronary artery disease	− 0.357	0.700	0.221	2.213	0.543
Chronic kidney disease (CKD ≥ 3)	1.841	6.302	0.571	69.524	0.133
Resuscitation	1.655	5.232	1.840	14.877	0.002
Chest trauma	0.501	1.650	0.914	2.981	0.097
Hemoglobin per g/dl	− 0.105	0.086	0.775	1.046	0.169

*ISS *injury severity score, *CKD *chronic kidney disease

^*^relative duration of hypotension < 65 mmHg per 10%

### Multivariate logistic regression—secondary endpoints

Results of multivariate logistic regression analysis for in-hospital death, acute kidney injury, and MACE are presented in Tables 3, 4 and 5. Adjusted OR of hypotension duration was 1.23 [95% CI 1.05–1.44] for in-hospital death and 1.0 [95% CI 0.85–1.18] for acute kidney injury, respectively. Hypotension duration was independently associated with MACE (OR 1.22 [95% CI 1.04–1.44]).

**Table 3:** Multivariate binary logistic regression model for hypotension and in-hospital death

Variable	Regression coefficient	Odds ratio	95% confidence interval		*p*-value
			Lower	Upper	
Hypotension*	0.206	1.228	1.045	1.444	0.013
Age per year	0.054	1.056	1.035	1.077	< 0.001
Sex	− 0.375	0.687	0.318	1.484	0.339
ISS-score	0.051	1.052	1.019	1.087	0.002
Coronary artery disease	0.140	1.151	0.335	3.948	0.823
Chronic kidney disease (CKD ≥ 3)	− 1.869	0.154	0.007	3.286	0.231
Resuscitation	2.318	10.159	3.579	28.837	< 0.001
Thorax trauma	− 0.856	0.425	0.202	0.894	0.024
Hemoglobin per g/dl	− 0.235	0.791	0.679	0.922	0.003

*ISS* injury severity score, *CKD* chronic kidney disease

^*^relative duration of hypotension < 65 mmHg per 10%

**Table 4:** Multivariate binary logistic regression model for hypotension and acute kidney injury

Variable	Regression coefficient	Odds ratio	95% confidence interval		*p*-value
			Lower	Upper	
Hypotension*	− 0.002	0.998	0.845	1.179	0.979
Age per year	0.035	1.035	1.014	1.057	0.001
Sex	− 0.930	0.395	0.146	1.069	0.067
ISS-score	0.038	1.039	1.007	1.072	0.015
Coronary artery disease	− 0.559	0.572	0.139	2.357	0.439
Chronic kidney disease (CKD ≥ 3)	0.114	1.120	0.097	12.900	0.927
Resuscitation	1.245	3.473	1.325	9.099	0.011
Hemoglobin per g/dl	− 0.051	0.951	0.813	1.112	0.528

*ISS* injury severity score, *CKD* chronic kidney disease

^*^relative duration of hypotension < 65 mmHg per 10%

**Table 5:** Multivariate binary logistic regression model for hypotension and MACE

Variable	Regression coefficient	Odds ratio	95% confidence interval		*p*-value
			Lower	Upper	
Hypotension*	0.201	1.223	1.041	1.438	0.014
Age per year	0.026	1.026	1.004	1.049	0.021
Sex	− 1.134	0.322	0.096	1.078	0.066
ISS-score	0.005	1.005	0.969	1.041	0.801
Coronary artery disease	− 0.194	0.824	0.182	3.729	0.802
Chronic kidney disease (CKD ≥ 3)	− 19.218	0.000	0.000	na	0.999
Resuscitation	1.124	3.078	1.142	8.300	0.026
Thorax trauma	0.530	1.699	0.694	4.164	0.246
Hemoglobin per g/dl	− 0.045	0.956	0.805	1.136	0.608

*MACE* major adverse cardiac events, *ISS* injury severity score, *CKD* chronic kidney disease

^*^relative duration of hypotension < 65 mmHg per 10%

### Additional results of sensitivity analyses

Multivariate logistic regression analysis for hypotension (relative duration of MAP < 65 mmHg) and myocardial injury (based on peak hsTnT within the first 72 h) revealed an OR of 1.25 [95% CI 1.005–1.557] (Table S1). Multivariate logistic regression analysis for hypotension (relative duration of Sys < 90 mmHg) and myocardial injury (at presentation) revealed an OR of 1.13 [95% CI 0.975–1.307] (Table S2). Multivariate regression analysis for hypotension (relative duration of Sys < 90 mmHg) and myocardial injury (based on peak hsTnT within the first 72 h) revealed an OR of 1.16 [95% CI 0.922–1.461] (Table S3). Multivariate regression analysis for hypotension (absolute duration of MAP < 65 mmHg) and myocardial injury (at presentation) showed an OR of 1.032 [95% CI 1.01–1.055] (Table S4). Multivariate regression analysis for hypotension (absolute duration of MAP < 65 mmHg) and myocardial injury (based on peak hsTnT within the first 72 h) revealed an OR of 1.042 [95% CI 1.002–1.083] (Table S5). Multivariate regression analysis for hypotension (absolute duration of Sys < 90 mmHg) and myocardial injury (at presentation) revealed an OR of 1.031 [95% CI 1.005–1.057] (Table S6). Multivariate regression analysis for hypotension (absolute duration of Sys < 90 mmHg) and myocardial injury (based on peak hsTnT within the first 72 h) showed an OR of 1.029 [95% CI 0.986–1.073] (Table S7). Results for multivariate regression analysis including preclinical variables can be found in supplementary tables S8–S9.

During review process, a further sensitivity analysis including only patients who received blood and/or coagulation products during resuscitation period was performed. In total, 124 patients (36.2%) received at least one unit of fresh frozen plasma, erythrocytes and/or any coagulation product (see Table 1) and 64 out of these 124 patients (51.6%) presented with myocardial injury. This analysis showed an independent association between hypotension (defined as MAP < 65 mmHg) and myocardial injury (OR 1.21, 95% CI 1.03–1.43, *P* = 0.023). The corresponding table can be found in the supplements (see Table S10).

## Discussion

The present study indicates that the duration of hypotension during ED resuscitation period is independently associated with the incidence of myocardial injury in patients with severe trauma. In addition, the duration of hypotension is independently associated with MACE and in-hospital mortality.

In the non-cardiac surgery setting, several large observational cohort studies already reported an association between hypotension and myocardial injury. Van Waes and colleagues analyzed a cohort of 890 prospective vascular surgery patients and found out that a decrease of 40% from preoperatively measured mean arterial blood pressure was associated with postoperative myocardial injury when the duration of hypotension was longer than 30 min (relative risk = 1.8 with a 99% confidence interval of 1.2–2.6) [14]. A large retrospective analysis of more than 53,000 non-cardiac surgery patients supports these findings and showed that mean arterial pressure < 65 mmHg was significantly related to both myocardial and acute kidney injury [4]. A further study by Abbott and coworkers investigated the relationship between intraoperative heart rate and systolic blood pressure with myocardial injury [15, 16]. This secondary analysis of the VISION study revealed that low blood pressure defined as systolic blood pressure < 100 mmHg with a co-existing elevated heart rate > 100 beats per minute was more strongly associated with myocardial injury compared with decreased systolic blood pressure < 100 mmHg alone (OR 1.42 [95% CI 1.15–1.76] versus OR 1.20 [95% CI 1.03–1.40]) [15].

The existing evidence in this context resulted in a consensus statement on intraoperative blood pressure for elective surgery [3] which summarizes that during adult non-cardiac surgery, decreased systolic arterial pressure < 100 mmHg and MAP < 60–70 mmHg may be associated with organ injury, e.g. myocardial injury.

We performed multiple sensitivity analyses to better characterize the association between hypotension and myocardial injury. Hypotension, defined by the duration of MAP < 65 mmHg was always associated with myocardial injury (either at presentation or based on peak hsTnT within 72 h after trauma). However, regarding systolic blood pressure with a cutoff < 90 mmHg, only the absolute duration of hypotension was associated with myocardial injury at presentation. All other sensitivity analyses for Sys < 90 mmHg showed no significant association. This could be explained by various reasons. In terms of the analyses using peak hsTnT as endpoint, there was a relevant selection bias in the measurement of follow-up troponins. In addition, it may be possible that the measurement of systolic blood pressure was more inaccurate than the measurement of MAP in this cohort. In the future, it should be investigated whether this discrepancy between MAP and systolic blood pressure can be reproduced.

With regard to hypotension and outcome, patients with severe trauma represent a special cohort because guidelines advocate permissive hypotension for bleeding control before surgical treatment is available. In severely injured patients with active bleeding, current guidelines recommend maintaining mean arterial pressure around 65 mmHg [17]. In line with these recommendations, our findings suggest that protracted hypotension < 65 mmHg is associated with myocardial injury and other adverse events.

Next to hypotension, various other mechanisms of myocardial injury in severe trauma patients have been proposed. One frequently postulated pathomechanism is direct mechanical trauma, causing cardiac contusion or “bruising” with consecutive cell death [18, 19]. However, recent studies indicated that troponin is also elevated in patients without blunt cardiac chest trauma. Edouard et al. investigated 17 trauma patients without chest trauma and could reveal significantly increased troponin levels in this cohort [20]. In several experimental trauma models, myocardial injury followed ischemia–reperfusion injury, inflammation and hemorrhagic shock [21–23] and it was interpreted to result from physiological stress. Martin et al. retrospectively investigated 1081 trauma patients admitted to the ICU. Severe chest trauma (defined as AIS ≥ 3) was not independently associated with increased troponin levels, and the authors concluded physiological stress played an essential role in the pathogenesis of myocardial injury in severe trauma patients [12]. Moreover, they identified that hypotension was more common in the group with high troponin levels, however, without quantifying the strength of the association or its independence.

The finding of the association between hypotension and in-hospital mortality in the present study is in line with previous data from a large cohort. Kim et al. retrospectively investigated 17,406 patients and could show that hypotension was associated with increased mortality in patients with trauma [24].

### Strengths and limitations

A strength of this study is that we investigated a broad cohort of patients with severe trauma. Especially, we included all types of injuries taking into account mechanisms of troponin release other than chest trauma. Second, although we performed a retrospective analysis, our data are based on a prospectively constituted database which ensures high data quality. This is also applicable for blood pressure values which were recorded permanently and automatically. On the other hand, our broad inclusion criteria could also be seen as a limitation as the cohort of patients in whom permissive hypotension is recommended is narrow. Due to the retrospective nature of the study, it is not possible for us to comprehend which patients really received permissive hypotension with the aim to control bleeding and which patients were hypotensive because of other reasons, e.g. induction of anesthesia.

There are further limitations to the present study: we could only analyze blood pressure values in the resuscitation room as pre-hospital documentation was not available. Thus, blood pressure and troponin measurements were performed simultaneously and hypotension in the course of resuscitation period may have been influenced by events and/or interventions after hsTnT measurement. However, there were several considerations serving as a basis for the decision to choose initial hsTnT as the primary endpoint of this analysis: first, in contrast to follow-up troponin measurements, baseline troponin data were almost complete and as troponin is measured routinely in our resuscitation room, data could be regarded as representative without any selection bias. Second, our decision was based on the assumption that patients with a longer period of hypotension during resuscitation period may also have had hypotension before entering the hospital. Third, we analyzed relevant secondary endpoints that occurred after resuscitation period (MACE and mortality) that were also independently associated with hypotension which emphasizes our findings. Finally, we performed additional analyses using peak hsTnT values as endpoint. Although selection bias might be present, these data help to understand our results. It is important to mention that we conducted a retrospective study. However, most patient characteristics correspond to the current literature so that our cohort may be regarded as representative.

## Conclusions

In patients suffering from severe trauma, the relative duration of hypotension < 65 mmHg during resuscitation period is independently associated with myocardial injury. While permissive hypotension is crucial before bleeding is controlled, clinicians should be aware of its potential for organ injury and minimize the duration of severe hypotension. Future studies should investigate these findings in larger cohorts with a prospective design focusing on follow-up troponin measurements.

## Supplementary Information

Below is the link to the electronic supplementary material.

Supplementary file1 (DOCX 45 KB)

## Data Availability

The datasets generated during and/or analyzed during the current study are available from the first author on reasonable request.
