# Supplementary material for: Association between hypotension and myocardial injury in patients with severe trauma
Source: Eur J Trauma Emerg Surg. 2022 Aug 3;49(1):217–25. doi: 10.1007/s00068-022-02051-5 (PMC9925499; doi:10.1007/s00068-022-02051-5)
Supplement: Supplementary file 1 — Supplementary file1 (DOCX 45 KB) [file 68_2022_2051_MOESM1_ESM.docx]

| **Variable** | **Regression Coefficient** | **Odds Ratio** | **95% Confidence interval** | | **p-value** |
| --- | --- | --- | --- | --- | --- |
|  |  |  | **lower** | **upper** |  |
| Hypotension* | .224 | 1.251 | 1.005 | 1.557 | 0.045 |
| Age per year | .029 | 1.029 | 1.010 | 1.049 | .003 |
| Sex | -.842 | .431 | .194 | .957 | .039 |
| ISS-Score | .014 | 1.014 | .973 | 1.056 | .503 |
| Coronary artery disease | .061 | 1.062 | .195 | 5.795 | .944 |
| Chronic kidney disease (CKD ≥ 3) | 19.960 | 466172286.767 | .000 | . | .999 |
| Resuscitation | 2.714 | 15.095 | 1.716 | 132.760 | .014 |
| Chest trauma | .493 | 1.637 | .787 | 3.405 | .188 |
| Hemoglobin per g/dl | -.201 | .818 | .671 | .998 | .047 |

**Table S1: Multivariate logistic regression analysis for hypotension (relative duration of MAP < 65mmHg) and myocardial injury (based on peak hs-TnT within the first 72h)**

*relative duration of hypotension MAP<65mmHg per 10%; ISS=Injury Severity Score, CKD = Chronic Kidney Disease

**Table S2: Multivariate logistic regression analysis for hypotension (relative duration of Sys < 90mmHg) and myocardial injury (at presentation)**

| **Variable** | **Regression Coefficient** | **Odds Ratio** | **95% Confidence interval** | | **p-value** |
| --- | --- | --- | --- | --- | --- |
|  |  |  | **lower** | **upper** |  |
| Hypotension* | .121 | 1.129 | 0.975 | 1.307 | 0.104 |
| Age per year | .030 | 1.031 | 1.017 | 1.044 | .000 |
| Sex | -.100 | .905 | .503 | 1.626 | .737 |
| ISS-Score | .023 | 1.023 | .999 | 1.049 | .064 |
| Coronary artery disease | -.193 | .825 | .310 | 2.195 | .700 |
| Chronic kidney disease (CKD ≥ 3) | 2.081 | 8.016 | .822 | 78.204 | .073 |
| Resuscitation | 1.815 | 6.143 | 2.432 | 15.518 | .000 |
| Chest trauma | .341 | 1.406 | .826 | 2.394 | .209 |
| Hemoglobin per g/dl | -.159 | .853 | .759 | .960 | .008 |

* relative duration of hypotension (systolic arterial pressure <90mmHg per 10%); ISS=Injury Severity Score, CKD = Chronic Kidney Disease

**Table S3: Multivariate logistic regression analysis for hypotension (relative duration of Sys < 90mmHg) and myocardial injury (based on peak hs-TnT within the first 72h)**

| **Variable** | **Regression Coefficient** | **Odds Ratio** | **95% Confidence interval** | | **p-value** |
| --- | --- | --- | --- | --- | --- |
|  |  |  | **lower** | **upper** |  |
| Hypotension* | 0.149 | 1.160 | 0.922 | 1.461 | 0.206 |
| Age per year | .028 | .411 | 1.010 | 1.048 | .003 |
| Sex | -.889 | 1.020 | .187 | .906 | .027 |
| ISS-Score | .020 | 1.040 | .980 | 1.061 | .335 |
| Coronary artery disease | .039 | 420427968.717 | .188 | 5.754 | .965 |
| Chronic kidney disease (CKD ≥ 3) | 19.857 | 15.035 | .000 | . | .999 |
| Resuscitation | 2.710 | 1.648 | 1.719 | 131.469 | .014 |
| Chest trauma | .499 | .803 | .802 | 3.384 | .174 |
| Hemoglobin per g/dl | -.220 | 1.160 | .658 | .978 | .029 |

* relative duration of hypotension (systolic arterial pressure <90mmHg per 10%); ISS=Injury Severity Score, CKD = Chronic Kidney Disease

**Table S4: Multivariate logistic regression analysis for hypotension (absolute duration of MAP < 65mmHg) and myocardial injury (at presentation)**

| **Variable** | **Regression Coefficient** | **Odds Ratio** | **95% Confidence interval** | | **p-value** |
| --- | --- | --- | --- | --- | --- |
|  |  |  | **lower** | **upper** |  |
| Hypotension* | 0.032 | 1.032 | 1.010 | 1.055 | 0.004 |
| Age per year | .031 | 1.031 | 1.017 | 1.045 | .000 |
| Sex | -.050 | .952 | .526 | 1.721 | .870 |
| ISS-Score | .017 | 1.017 | .992 | 1.042 | .185 |
| Coronary artery disease | -.223 | .800 | .295 | 2.167 | .660 |
| Chronic kidney disease (CKD ≥ 3) | 2.183 | 8.872 | .909 | 86.617 | .060 |
| Resuscitation | 1.734 | 5.662 | 2.201 | 14.565 | .000 |
| Chest trauma | .342 | 1.407 | .821 | 2.413 | .214 |
| Hemoglobin per g/dl | -.149 | .862 | .765 | .971 | .015 |

* absolute duration of hypotension MAP<65mmHg per 10%; ISS=Injury Severity Score, CKD = Chronic Kidney Disease

**Table S5: Multivariate logistic regression analysis for hypotension (absolute duration of MAP < 65mmHg) and myocardial injury (based on peak hs-TnT within the first 72h)**

| **Variable** | **Regression Coefficient** | **Odds Ratio** | **95% Confidence interval** | | **p-value** |
| --- | --- | --- | --- | --- | --- |
|  |  |  | **lower** | **upper** |  |
| Hypotension* | 0.041 | 1.042 | 1.002 | 1.083 | 0.041 |
| Age per year | .028 | 1.028 | 1.009 | 1.048 | .003 |
| Sex | -.875 | .417 | .187 | .927 | .032 |
| ISS-Score | .015 | 1.015 | .974 | 1.058 | .470 |
| Coronary artery disease | .033 | 1.034 | .186 | 5.747 | .970 |
| Chronic kidney disease (CKD ≥ 3) | 19.960 | 465924910.77 | .000 | . | .999 |
| Resuscitation | 2.700 | 14.886 | 1.690 | 131.107 | .015 |
| Chest trauma | .469 | 1.598 | .766 | 3.333 | .212 |
| Hemoglobin per g/dl | -.198 | .821 | .673 | 1.001 | .051 |

* absolute duration of hypotension MAP<65mmHg per 10%; ISS=Injury Severity Score, CKD = Chronic Kidney Disease

**Table S6: Multivariate logistic regression analysis for hypotension (absolute duration of Sys < 90mmHg) and myocardial injury (at presentation)**

| **Variable** | **Regression Coefficient** | **Odds Ratio** | **95% Confidence interval** | | **p-value** |
| --- | --- | --- | --- | --- | --- |
|  |  |  | **lower** | **upper** |  |
| Hypotension* | 0.030 | 1.031 | 1.005 | 1.057 | 0.019 |
| Age per year | .031 | 1.031 | 1.018 | 1.045 | .000 |
| Sex | -.100 | .904 | .503 | 1.627 | .737 |
| ISS-Score | .021 | 1.021 | .997 | 1.046 | .091 |
| Coronary artery disease | -.239 | .788 | .293 | 2.115 | .636 |
| Chronic kidney disease (CKD ≥ 3) | 2.112 | 8.265 | .844 | 80.901 | .070 |
| Resuscitation | 1.738 | 5.683 | 2.230 | 14.485 | .000 |
| Chest trauma | .312 | 1.366 | .799 | 2.336 | .254 |
| Hemoglobin per g/dl | -.155 | .856 | .761 | .963 | .010 |

* absolute duration of hypotension (systolic arterial pressure <90 mmHg per 10%); ISS=Injury Severity Score, CKD = Chronic Kidney Disease

**Table S7: Multivariate logistic regression analysis for hypotension (absolute duration of Sys < 90mmHg) and myocardial injury (based on peak hs-TnT within the first 72h)**

| **Variable** | **Regression Coefficient** | **Odds Ratio** | **95% Confidence interval** | | **p-value** |
| --- | --- | --- | --- | --- | --- |
|  |  |  | **lower** | **upper** |  |
| Hypotension* | 0.028 | 1.029 | 0.986 | 1.073 | 0.196 |
| Age per year | .028 | 1.028 | 1.009 | 1.047 | .003 |
| Sex | -.905 | .405 | .184 | .892 | .025 |
| ISS-Score | .021 | 1.021 | .981 | 1.062 | .313 |
| Coronary artery disease | .012 | 1.012 | .180 | 5.679 | .989 |
| Chronic kidney disease (CKD ≥ 3) | 19.851 | 417890283.22 | .000 | . | .999 |
| Resuscitation | 2.692 | 14.757 | 1.686 | 129.192 | .015 |
| Chest trauma | .460 | 1.584 | .769 | 3.261 | .212 |
| Hemoglobin per g/dl | -.217 | .805 | .661 | .981 | .032 |

*absolute duration of hypotension (systolic arterial pressure <90 mmHg per 10%); ISS=Injury Severity Score, CKD = Chronic Kidney Disease

**Table S8: Multivariate logistic regression analysis for the association between hypotension (relative duration of MAP < 65mmHg) and myocardial injury (at presentation) including only preclinical variables**

| **Variable** | **Regression Coefficient** | **Odds Ratio** | **95% Confidence interval** | | **p-value** |
| --- | --- | --- | --- | --- | --- |
|  |  |  | **lower** | **upper** |  |
| Hypotension* | .223 | 1.249 | 1.042 | 1.498 | .016 |
| Glscow coma scale | -.027 | .973 | .896 | 1.057 | .522 |
| Systolic blood pressure | .000 | 1.000 | .992 | 1.009 | .921 |
| Heart rate | .004 | 1.004 | .990 | 1.017 | .611 |
| Respiratory rate | -.046 | .955 | .896 | 1.018 | .156 |
| SpO2 | -.025 | .975 | .946 | 1.006 | .110 |
| Therapy with Cristalloids | -.001 | .999 | .998 | 1.000 | .038 |
| Therapy with Colloids | -.002 | .998 | .995 | 1.002 | .406 |
| Resuscitation | .946 | 2.576 | .455 | 14.587 | .285 |
| Vasopressor therapy | .684 | 1.981 | .788 | 4.982 | .146 |
| Chest drainage | .719 | 2.052 | .377 | 11.174 | .406 |
| Tranexamic acid | -2.211 | .110 | .013 | .892 | .039 |
| Sedative medication | -.665 | .514 | .225 | 1.176 | .115 |
| Intubation | .852 | 2.344 | .929 | 5.912 | .071 |

*relative duration of hypotension MAP<65mmHg per 10%; SpO2: oxygen saturation;

**Table S9: Multivariate logistic regression analysis for the association between hypotension (relative duration of MAP < 65mmHg) and myocardial injury (based on peak hs-TnT within the first 72h) including only preclinical variables**

| **Variable** | **Regression Coefficient** | **Odds Ratio** | **95% Confidence interval** | | **p-value** |
| --- | --- | --- | --- | --- | --- |
|  |  |  | **lower** | **upper** |  |
| Hypotension* | .296 | 1.344 | 1.001 | 1.804 | .049 |
| Glscow coma scale | -.101 | .904 | .803 | 1.018 | .096 |
| Systolic blood pressure | .014 | 1.015 | 1.000 | 1.030 | .055 |
| Heart rate | -.005 | .995 | .976 | 1.014 | .578 |
| Respiratory rate | .008 | 1.008 | .923 | 1.101 | .858 |
| SpO2 | -.005 | .995 | .952 | 1.040 | .829 |
| Therapy with Cristalloids | .000 | 1.000 | .999 | 1.001 | .563 |
| Therapy with Colloids | -.002 | .998 | .994 | 1.002 | .310 |
| Resuscitation | 20.097 | 534577703.52 | .000 | . | .999 |
| Vasopressor therapy | .921 | 2.511 | .533 | 11.835 | .245 |
| Chest drainage | -.328 | .720 | .070 | 7.455 | .783 |
| Tranexamic acid | -1.327 | .265 | .020 | 3.486 | .313 |
| Sedative medication | .893 | 2.443 | .741 | 8.057 | .142 |
| Intubation | -1.454 | .234 | .064 | .851 | .027 |

*relative duration of hypotension MAP<65mmHg per 10%; SpO2: oxygen saturation;

**Table S10: Multivariate logistic regression analysis for hypotension (relative duration of MAP < 65mmHg) and myocardial injury (at presentation) in patients who received blood and / or coagaulation products (n=124)**

| **Variable** | **Regression Coefficient** | **Odds Ratio** | **95% Confidence interval** | | **p-value** |
| --- | --- | --- | --- | --- | --- |
|  |  |  | **lower** | **upper** |  |
| Hypotension* | .192 | 1.211 | 1.027 | 1.430 | 0.023 |
| Age per year | .012 | 1.012 | 0.992 | 1.032 | .244 |
| Sex | -.553 | .575 | .240 | 1.378 | .214 |
| ISS-Score | .030 | 1.031 | .995 | 1.068 | .092 |
| Coronary artery disease | 1.237 | 3.446 | .562 | 21.116 | .181 |
| Chest trauma | 1.639 | 0.194 | n.a. | n.a. | .065 |

*relative duration of hypotension MAP<65mmHg per 10%; ISS=Injury Severity Score, n.a.= not applicable
